# Electrospinning Drug-Loaded Alginate-Based Nanofibers towards Developing a Drug Release Rate Catalog

**DOI:** 10.3390/polym14142773

**Published:** 2022-07-06

**Authors:** Kathryn E. Penton, Zachary Kinler, Amber Davis, Joshua A. Spiva, Sharon K. Hamilton

**Affiliations:** 1 Department of Chemistry and Physics, Delta State University, Cleveland, MS 38733, USA; kathryn.e.penton@vanderbilt.edu (K.E.P.); kinlerzachary@gmail.com (Z.K.); amberwilson1319@gmail.com (A.D.); 2 Department of Chemistry and Physics, Ouachita Baptist University, Arkadelphia, AR 71998, USA; joshua.spiva01@gmail.com

**Keywords:** biomaterials, naturally-occurring polymers, alginate, polysaccharide, electrospinning

## Abstract

Electrospinning natural polymers represents a developing interest in the field of biomaterials. Electrospun nanofibers have been shown to facilitate tissue regeneration and emulate body tissue, making them ideal for modern biomedical applications. These water-soluble natural polymers including alginate, have also shown promise as drug delivery vehicles. However, many biopolymers including alginate are inherently charged, making the formation of nanofibers difficult. To better understand the potential of natural polymer-based fibers in drug delivery applications, fiber formulations and drug loading concentrations of alginate-based scaffolds were investigated. It was found electrospinning poly(vinyl alcohol) with alginate facilitated fiber formation while the co-polymer agarose showed minor improvement in terms of alginate electrospinnability. Once uniform fibers were formed, the antibiotic ciprofloxacin was added into the polymer electrospinning solution to yield drug-loaded nanofibers. These optimized parameters coupled with small molecule release rate data from the drug-loaded, alginate-based fibers have been used to establish a catalog of small molecule release profiles. In the future, this catalog will be further expanded to include drug release rate data from other innately charged natural polymer-based fibers such as chitosan. It is anticipated that the cataloged profiles can be applied in the further development of biomaterials used in drug delivery.

## 1. Introduction

Biomaterials research has recently been investigating natural polymers due to their biocompatibility and nontoxic nature. One of the natural polymers in use is alginate (Alg), an anionic polysaccharide found in brown algae. Alginate-based products are particularly suited for biomedical applications since they are biocompatible, biodegradable, and non-immunogenic. Such constructs have been used in 2-D and 3-D cell culture as well as in drug delivery and wound healing applications [1,2,3,4,5,6].

Electrospinning solutions of polymers such as alginate allows for the creation of non-woven, fibrous scaffolds that resemble the architecture of the extracellular matrix-a useful characteristic for wound healing dressings and tissue regeneration scaffolds [7,8]. With fiber diameters ranging from the nanometer to microscale, electrospun scaffolds possess a high surface-to-volume ratio, tunable porosity, and flexibility to conform to a variety of sizes and shapes [9]. Additionally, electrospinning represents an attractive approach for polymer biomaterials processing with the opportunity to control scaffold composition to combine desired compounds, properties, and functionalities [10,11,12]. These advantages allow electrospun scaffolds to address specific application challenges in wound healing, tissue engineering, and delivery matrices [9,13,14].

However, little research has been published on the controlled delivery of drugs using natural polymeric dressings, especially nanofibers [7]. Local delivery of drugs to a site should be sustained for at least one week to improve the overall effectiveness of the drug therapy and reduce the frequency of dressing replacements [15,16,17]. The fine diameter and highly porous structure of nanofiber scaffolds help drug molecules efficiently diffuse out of the structure; though, a burst release is often observed when therapeutic-loaded nanofibers have been investigated as drug delivery vehicles [15,18,19,20].

The innate charges from functional groups on natural polymers make these compounds difficult to electrospin. To overcome this inert charge, co-polymers with similar intermolecular forces as the natural polymers are incorporated into the spinning solution to aid fiber formation [8,15,21,22,23]. Blending of polymers for electrospinning provides a straightforward method to combine different bioactivities, properties, and characteristics for biomedical applications [9,24,25]. To date, there are few reports investigating the impact of these co-polymers or the innate charges in the biopolymers on the drug release capabilities of natural polymer electrospun fibers. Combining alginate with poly(vinyl alcohol) or agarose via electrospinning should capitalize on the properties of both biocompatible materials in each fiber type to create a unique drug release profile for each scaffold.

Here we report our initial findings on the impact of co-polymers and innate polymer charges on the small molecules release rate of natural polymer-based scaffolds. Our goal was to utilize two co-polymers, poly(vinyl alcohol) (PVA) and agarose (Ag) in conjunction with alginate to create alginate–based nanofibers with minimal beading and polymer webbing to provide a uniform scaffold for loading the small molecule drug ciprofloxacin (Cipro). These co–polymers were selected to compare the impact of a synthetic versus natural co-polymer. The overall aims of this project were: (1) to develop and analyze alginate-based electrospun fibers; and (2) to study the release rate of a small molecule from negatively charged, alginate-based nanofibers. These results will eventually be compared to drug release rates from positively charged, natural polymer-based fibers to further analyze the impact of co-polymers and innate polymer charges on small molecule therapeutic release rates from nanofiber scaffolds.

## 2. Materials and Methods

### 2.1. Materials

Sodium alginate powder (Alginic acid sodium salt (Alg) from brown algae, medium viscosity, MW 80,000–120,000, (Sigma-Aldrich, St. Louis, MO, USA)), poly(vinyl alcohol) (PVA) powder 87–89% hydrolyzed, high molecular weight (Sigma-Aldrich, St. Louis, MO, USA), agarose powder (Agarose (Ag) Type III–A, High EEO) (Sigma-Aldrich, St. Louis, MO, USA), and ciprofloxacin powder (Ciprofloxacin (Cipro), 98%, Ciprofloxacin hydrochloride hydrate, 98%) (Alfa Aesar, Haverhill, MA, USA) were all obtained through commercial suppliers and used without purification or alteration unless otherwise noted.

### 2.2. Creation of Alginate-Based Nanofibers

#### 2.2.1. Preparation of Alginate and PVA (Alg-PVA) Electrospinning Solution

Sodium alginate powder (2.0–2.5 g) was dissolved in 10 mL dH_2_O to make a stock solution of the desired concentration and stirred until homogeneous. PVA powder (10.0 g) was dissolved in 10 mL of dH_2_O to make a stock solution of the desired concentration and heated at 80 °C until thoroughly dissolved. The PVA stock solution was brought to room temperature before further use.

The desired volumes of alginate and PVA solutions were micropipetted into one vial to create a 10 mL electrospinning solution. The combined polymer solution was then placed on a shaker plate for approximately 1 h to ensure the two components were properly incorporated before electrospinning.

#### 2.2.2. Preparation of Alginate and Agarose (Alg-Ag) Electrospinning Solution

Sodium alginate powder was prepared as previously mentioned. Agarose powder (0.5–1.0 g) was dissolved in 10 mL dH_2_O to make stock solutions of desired concentrations. The solutions were stirred and heated at 95 °C until thoroughly dissolved. Agarose solutions were allowed to cool before incorporation into alginate solutions. The Alg-Ag solutions were prepared as described above for the Alg-PVA solutions.

#### 2.2.3. Preparation of Cipro-Loaded Alginate-Based Electrospinning Solution

Sodium alginate, PVA, and agarose solutions were prepared as previously mentioned. Ciprofloxacin powder (3.0–5.0 g) was dissolved in the respective polymer solution (10 mL) and stirred overnight.

### 2.3. Electrospinning Alginate-Based Nanofibers

A Spraybase^®^ (Spraybase, Dublin, Ireland) 30 kV electrospinner and a laptop computer (Windows 10, Acer Inc., New Taipei City, Taiwan) was dedicated to this system for equipment control, data collection, and syringe pump program design (SyringePumpPro V1 Version:1.6.4.7). Electrospinning solutions were initially spun at 5.8, 7.5, and 9.8 μL/min. Electrospinning volumes and voltages fluctuated dependent on sample however the height from the collector plate to the needle was constant at 100 mm unless otherwise stated.

### 2.4. Analysis of Nanofibers

A JEOL, Ltd. (Akishima, Tokyo, Japan) JSM-6010LA variable pressure scanning electron microscope (SEM) was used at 10 and 15 kV for sample imaging and an EMS 550X Auto Sputter Coating Device with carbon coating attachment was used for gold sputter coating samples before imaging. SEM micrograph images of fibers were analyzed using Zeiss 3.4 ZEN Blue edition software (Carl Zeiss AG, Oberkochen, Germany). Briefly, the diameters of 40 fibers per fiber type were measured and averaged and the standard deviation of each fiber type calculated using Excel^®^ (V. 16.6.1, Microsoft, Redmond, WA, USA). A paired *t*-test at 95% confidence level was conducted to determine if the loading of ciprofloxacin into nanofibers significantly impacted the diameter of the resulting fibers.

### 2.5. Release Studies of Drug-Loaded Alginate-Based Nanofibers

The drug-loaded alginate-based nanofibers were cut into 1.5 centimeters (cm) × 1.5 cm samples and placed into 20 mL of phosphate-buffered saline solution (PBS) at pH = 7.4 and incubated at 37 °C for 14 days [15,26,27]. These conditions were selected to mimic natural body temperature and pH levels to observe drug release from the fiber mats in near physiological settings. Every 24 h, 10 μL aliquot was collected and replaced with fresh PBS buffer. The amount of Cipro released from the drug-loaded mats was determined via ultraviolet-visible spectroscopy (UV-Vis, NanoDrop One^C^, Thermo Fisher Scientific, Waltham, MA, USA) at a wavelength (λ**_max_**) of 271 nanometers (nm). The collected samples were analyzed by UV-Vis absorption measurements taken in triplicate and compared to a standard calibration curve made from aqueous Cipro standard solutions to determine the release concentration of Cipro in each sample. A ***p***-test at 95% confidence level was conducted to determine if the ciprofloxacin release rates from the drug-loaded scaffolds was significantly different than the control, non-loaded samples. These data provided the small molecule release concentration from the nanofiber mat as a function of time.

## 3. Results

### 3.1. Alg-PVA Nanofibers

**Figure 1:**
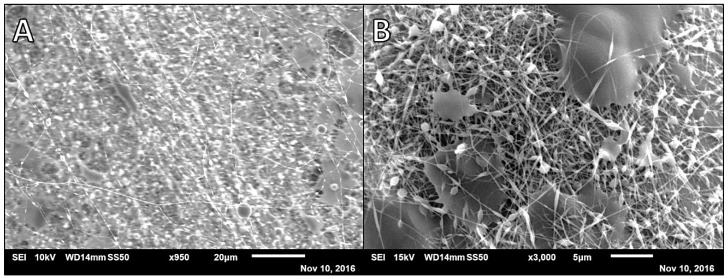
Scanning electron microscopy (SEM) micrographs of 7:3 2% alginate (Alg):10% poly(vinyl alcohol (PVA) at different magnifications; average diameter = 157.9 nm, *SD* = 43.1, *n* = 40. (**A**) × 950 and (**B**) × 3000 at 5.8 µL/min, 18.5 kV, 90 mm, and 2 mL dispensed (disp).

Preliminary efforts to prepare alginate-based fibers began with using the synthetic polymer PVA as a co-polymer to facilitate fiber formation. To make alginate the major component of the fiber mats, initial efforts to prepare alginate and PVA fibers began at a 7:3 ratio of 2% weight per volume (*w/v*) alginate to 10% (*w/v*) PVA (based on previously unpublished data). SEM analysis of this sample showed heavy polymer beading on the fibers as well as webbing of fibers together by polymer that did not effectively spin (Figure 1). These fibers had an average diameter of 157.9 nm (*SD* = 43.1). The inconsistent fiber architecture could convolute drug release results and future studies of the mat degradation and thus additional fiber formulations and electrospinning parameters were investigated prior to drug loading.

**Figure 2:**
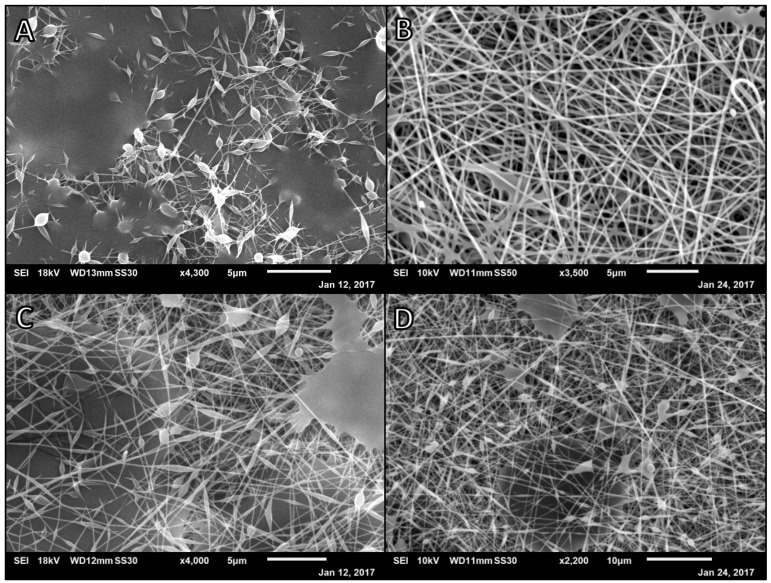
SEM micrographs of 2.5% Alg:10% PVA nanofibers at 7.5 µL/min, 100 mm, and different ratios. (**A**) 8:2 2.5% Alg:10%PVA at 20 kV and 0.99 mL disp.; (**B**) 7:3 2.5% Alg:10% PVA at 25 kV and 2 mL disp.; (**C**) 1:1 2.5% Alg:10% PVA at 30 kV and 2.5 mL disp.; (**D**) 2:8 2.5% Alg:10% PVA at 25 kV and 2 mL disp.

Subsequently, the alginate percentage was increased to 2.5% (*w/v*) and different polymer ratios investigated with all samples being spun at a rate of 7.5 μL/min with a target distance of 100 mm (Figure 2) [28,29]. Polymer compositions ranged from a majority natural polymer to a majority synthetic polymer. A ratio of 8:2 of alginate to PVA solutions spun at a lower voltage (20 kV) yielded fibers with extensive webbing to the point that fiber diameters were unable to be measured consistently (Figure 2A). A ratio of 7:3 of alginate to PVA solutions was spun at 25 kV and yielded fibers with an average diameter of 197.8 nm (*SD* = 44.3) with very little beading and some webbing (Figure 2B). An equal ratio of alginate to PVA solutions spun at 30 kV yielded a wide distribution of nanofiber diameters with beading and some polymer webbing connecting fibers (*M* = 157.7 nm, *SD* = 47.9, Figure 2C). A ratio of 2:8 of alginate to PVA solutions spun at 25 kV yielded wider fibers with smaller beads and less webbing (*M* = 189.5 nm, *SD* = 59.6, Figure 2D).

**Figure 3:**
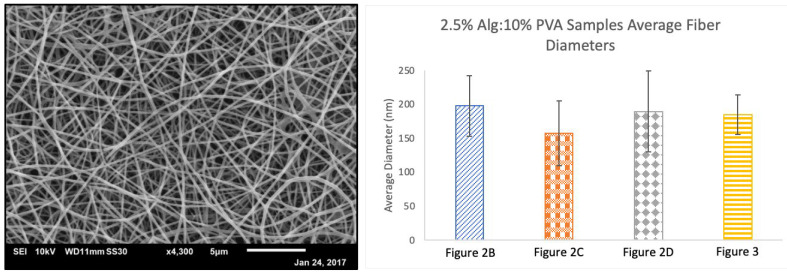
Left: SEM micrograph of Alg–PVA nanofibers. 3:7 2.5% Alg:10% PVA at 7.5 µL/min, 30 kV, 100 mm, and 2.5 mL disp. Right: Comparison of average Alg/PVA fiber diameters, *n* = 40. Each label corresponds to the referenced fiber formulation and spinning conditions described for the micrograph image of the fibers. All fibers formed spun at a rate of 7.5 µL/min, with a target distance of 100 mm: Figure 2B 7:3 2.5% Alg:10% PVA at 25 kV; Figure 2C 1:1 2.5% Alg:10% PVA at 30 kV; Figure 2D 2:8 2.5% Alg:10% PVA at 25 kV; Figure 3 3:7 2.5% Alg:10% PVA at 30 kV.

It was found that lowering the amount of alginate in the electrospinning solution and using a higher voltage yielded nanofibers with more consistent diameters without beading or webbing. This algins with the literature and common use of a co-polymer when electrospinning alginate [30,31,32]. A 3:7 ratio of 2.5% (*w/v*) alginate solution to 10% (*w/v*) PVA solution produced fibers with minimal to no beading or webbing when spun at a rate of 7.5 µL/min, with a 100 mm target distance, using 15 kV of electricity (*M* = 185.1 nm, *SD* = 29.4, Figure 3). These conditions were used as the finalized parameters for electrospinning alginate and PVA fibers and served as the initial formulation and spinning conditions for preparing drug–loaded alginate and PVA nanofibers. As the standard deviation of average nanofiber diameters decreased as the uniformity of nanofibers increased, we believe beading on the fibers resulted in the formation of fibers with inconsistent diameters.

### 3.2. Alg-Ag and Alginate, PVA, and Agarose (Alg-PVA-Ag) Nanofibers

**Figure 4:**
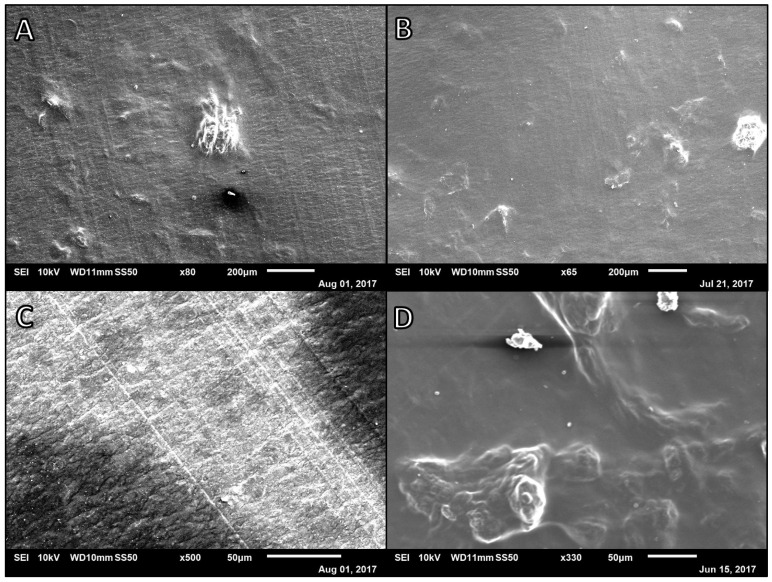
SEM micrographs of 1:1 2.5% Alg: agarose (Ag) samples at various concentrations of agarose spun with a target distance of 100 mm. (**A**) 0.5% Ag at 5.8 µL/min, 25 kV, and 1 mL disp.; (**B**) 0.5% Ag at 7.5 µL/min, 30 kV, and 0.2 mL disp.; (**C**) 0.75% Ag at 5.8 µL/min, 22 kV, and 1 mL disp.; and (**D**) 1% Ag at 7.5 µL/min, 19 kV, and 1 mL disp.

**Figure 5:**
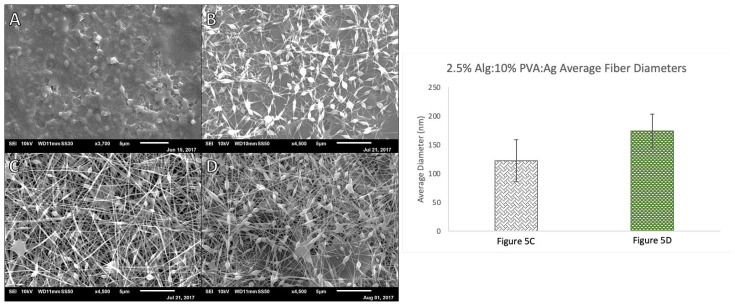
Left: SEM micrographs of Alg-PVA-Ag nanofibers at various ratios and concentrations at 7.5 µL/min and 100 mm. (**A**) 2:1:1 2.5% Alg:10% PVA:1% Ag at 19 kV and 0.5 mL disp.; (**B**) 2:1:1 2.5% Alg:10% PVA:0.5% Ag at 12 kV and 1.0 mL disp.; (**C**) 1:2:1 2.5% Alg:10% PVA:0.5% Ag at 22.8 kV and 1.0 mL disp.; (**D**) 1:2:2 2.5% Alg:10% PVA:0.5% Ag at 22 kV and 1.0 mL disp. Right: Comparison of average Alg:PVA:Ag fiber diameters, *n* = 40. Each label corresponds to the referenced fiber formulation and spinning conditions described for the micrograph image of the fibers. All fibers formed spun at a rate of 7.5 µL/min, with a target distance of 100 mm: Figure 5C 1:2:1 2.5% Alg:10% PVA:0.5% Ag at 22.8 kV; Figure 5D 1:2:2 2.5% Alg:10% PVA:0.5% Ag at 22 kV.

Next, the development of novel alginate fibers spun with natural co-polymer agarose was explored. Due to the low water solubility of agarose, lower agarose concentrations (0.5–1%, *w/v*) were used to prepare alginate and agarose samples. One to one ratios of alginate solution to 0.5%, 0.75%, or 1.0% (*w/v*) agarose were utilized. However, solutions of pure alginate and agarose did not yield fiber formation regardless of electrospinning rate or voltage. Micrograph images of samples from these spins revealed large aggregations of polymer or smoother polymer surfaces (Figure 4). As a result, PVA was used as a co-polymer in the alginate and agarose electrospinning solutions to help produce alginate and agarose–based nanofibers (Figure 5).

Alginate, PVA, and agarose samples were prepared using 2.5% (*w/v*) alginate, 0.5–1% (*w/v*) agarose, and 10% (*w/v*) PVA solutions (Figure 5). To have alginate be the major component of the fiber mats, initial efforts to prepare alginate, agarose, and PVA fibers began at a 2:1:1 ratio of 2.5% *w/v* Alg to 10% *w/v* PVA to 1% *w/v* Ag. This formulation yielded a sample with significant amounts of webbing and beading and distinct, individual fiber were distinguishable. For the next trial, the ratio of polymers was kept constant and the concentration of the agarose decreased to 0.5% *w/v*. This yielded less webbing in the sample; however, beading was prevalent and thus fiber diameters were unable to be measured for this formulation. Decreasing the amount of alginate in the electrospinning solution (1:2:1, Alg:PVA:Ag, Figure 5C) and increasing the voltage led to fiber formation with an average fiber diameter of 122.5 nm (*SD* = 36.7) yet there was still beads along the fibers. Increasing the amount of agarose in the formulation (1:2:2, Alg:PVA:Ag, Figure 5D) yielded thicker fibers with beading as well as webbing connecting fibers (*M* = 174.4 nm, *SD* = 29.3).

While electrospun alginate, PVA, and agarose fibers still have beads, the webbing on the samples has been greatly reduced. Optimal electrospinning conditions and fiber formulations have not yet been determined for alginate, PVA, and agarose fiber mats. The literature indicates that agarose is often structurally modified prior to electrospinning or that a co-polymer or organic solvents are required for agarose-based fibers to form [33,34,35]. In lieu of a polymer formulation that will yield uniform fibers, these novel, tri-component fibers were not utilized in drug release studies. There was concern that inconsistent beading and webbing on these fibers would result in inconsistent small molecule release rates as compared to uniform fibers. Since the goal of these studies is to establish a release rate catalog with data for each unique fiber formulation, it was determined that beading and webbing was not desired in the fiber scaffolds used to collect the release rate data.

### 3.3. Cipro-Loaded Alg-PVA Nanofibers

**Figure 6:**
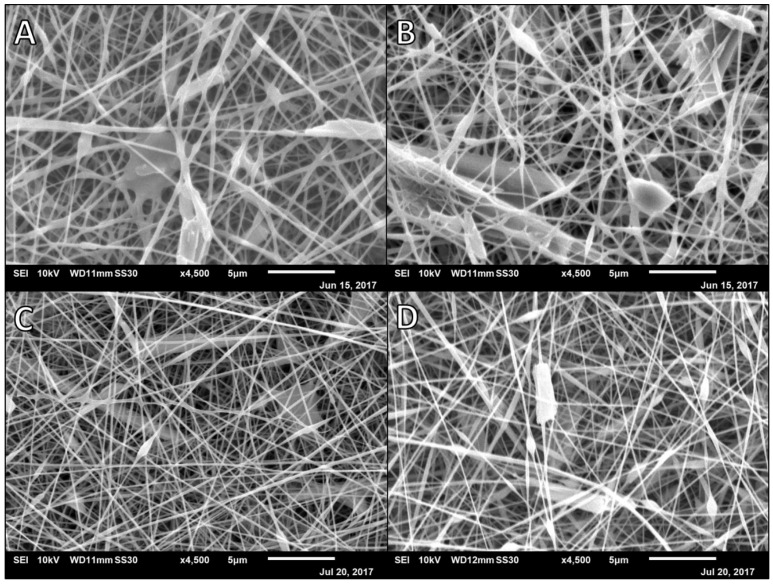
SEM micrographs of drug-loaded 3:7 2.5% Alg:10% PVA nanofibers at 100 mm, 1 mL disp. (**A**) 5% Ciprofloxacin (Cipro) at 7.5 µL/min and 19 kV; (**B**) 5% Cipro at 9.8 µL/min and 20 kV; (**C**) 4% Cipro at 7.5 µL/min and 20 kV; and (**D**) 4% Cipro at 9.8 µL/min and 15 kV.

The finalized parameter of a 3:7 ratio of 2.5% (*w/v*) alginate solution to 10% (*w/v*) PVA solution was used as the polymer electrospinning solution for all drug-loaded samples. Ciprofloxacin, an antibiotic used to treat infections was chosen as a model drug. Ciprofloxacin-containing polymer solutions were prepared as described by blending solid ciprofloxacin into the polymer solutions to the desired final antibiotic concentration. Fibers prepared from 5% (*w/v*) ciprofloxacin-loaded solution spun at a rate of 7.5 μL/min at 19 kV showed beading and some webbing between fibers with an average diameter of 206.7 nm (*SD* = 41.7). Increasing the dispensing rate and voltage to 9.8 μL/min and 20 kV, respectively, yielded thinner fibers with some beading but very little webbing (*M* = 178.7 nm, *SD* = 48.3). Lowering the ciprofloxacin concentration to 4% (*w/v*) yielded thinner fibers (*M* = 148.0 nm, *SD* = 34.8) with less beading and webbing in the samples when spun at a rate of 7.5 μL/min and 20 kV and thicker fibers (*M* = 183.7 nm, *SD* = 52.4) when spun at a rate of 9.8 μL/min at 15 kV (Figure 6).

**Figure 7:**
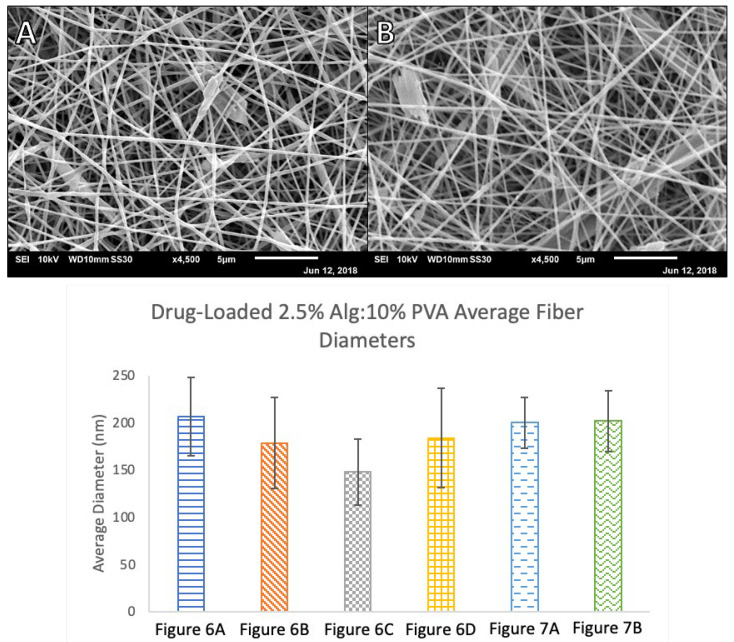
Top: SEM micrographs of lower-concentration drug-loaded 3:7 2.5% Alg:10% PVA nanofibers at 5.8 µL/min, 150 mm, 21 kV, and 1 mL disp. (**A**) 3.5% Cipro and (**B**) 3% Cipro. Bottom: Comparison of average drug-loaded 2.5% Alg:10% PVA fiber diameters, *n* = 40. Each label corresponds to the referenced fiber formulation and spinning conditions described for the micrograph image of the fibers. All fibers are 3:7 2.5% Alg:10% PVA. Figure 6A 5% Cipro at 7.5 µL/min, 100 mm, 19 kV; Figure 6B 5% Cipro at 9.8 µL/min, 100 mm, 20 kV; Figure 6C 4% Cipro at 7.5 µL/min, 100 mm, 20 kV; Figure 6D 4% Cipro at 9.8 µL/min, 100 mm, 15 kV; Figure 7A 3.5% Cipro at 5.8 µL/min, 150 mm, 21 kV; and Figure 7B 3% Cipro at 5.8 µL/min, 150 mm, 21 kV.

To further optimize scaffold uniformity, ciprofloxacin concentrations were lowered to 3.5 and 3% (*w/v*) while maintaining the same alginate and PVA concentrations and ratios (Figure 7). The 3.5% (*w/v*) ciprofloxacin solution produced nanofibers with an average diameter of 200.1 nm (*SD* = 26.9) and the 3% (*w/v*) ciprofloxacin solution produced fibers with an average diameter of 201.9 nm (*SD* = 32.3). It was found that non-loaded alginate and PVA fibers were not significantly different in size to the 4% and 5% (*w/v*) ciprofloxacin-loaded scaffolds. However, the 3% and 3.5% (*w/v*) ciprofloxacin-loaded mats were significantly larger in diameter than the non-loaded fibers. The calculated t-value at 95% confidence was 1.684 for this analysis. While the drug-containing polymer solution appeared to be homogenously mixed when it was electrospun, occasionally larger ciprofloxacin crystals were observed in the micrographs of ciprofloxacin-loaded fibers, particularly at higher drug concentrations. We believe this indicates that lower concentrations of ciprofloxacin were more effectively incorporated into the nanofibers resulting in thicker, more uniform fibers. This also accounts for the significant difference in the diameters of non-loaded nanofibers versus the 3% and 3.5% (*w/v*) ciprofloxacin-loaded fibers.

### 3.4. Release Studies on Drug-Loaded Alg-PVA Nanofibers

**Figure 8:**
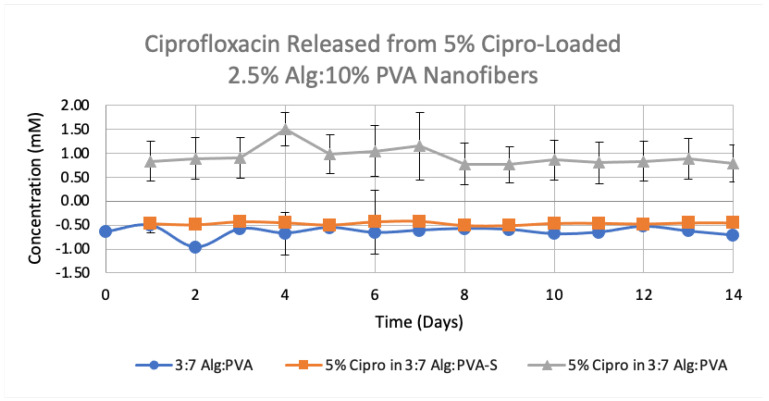
Comparison of average ciprofloxacin release over 14 days from two sizes of 5% Cipro-loaded 3:7 2.5% Alg:10% PVA nanofibers (*n* = 3, *p* ≤ 0.001). 5% Cipro in 3:7 Alg:PVA-S sample was 0.5 cm × 0.5 cm and 5% Cipro in 3:7 Alg: PVA sample was 1.5 cm × 1.5 cm. A standard deviation for each time point is given.

Upon successfully preparing uniform ciprofloxacin-loaded fibers, an initial drug release study was conducted comparing 5% (*w/v*) ciprofloxacin-loaded 3:7 2.5% (*w/v*) Alg:10% (*w/v*) PVA fibers to 3:7 2.5% (*w/v*) Alg:10% (*w/v*) PVA as the control (Figure 8). For this study, two different sizes of drug-loaded mats were used, 1.5 cm × 1.5 cm and 0.5 cm × 0.5 cm (5% Cipro in 3:7 Alg:PVA-S). The 1.5 cm × 1.5 cm ciprofloxacin-loaded sample showed a significant release of ciprofloxacin compared to the control and the smaller sized drug-loaded sample which did not show much release of ciprofloxacin (*p*-test, 95% confidence).

**Figure 9:**
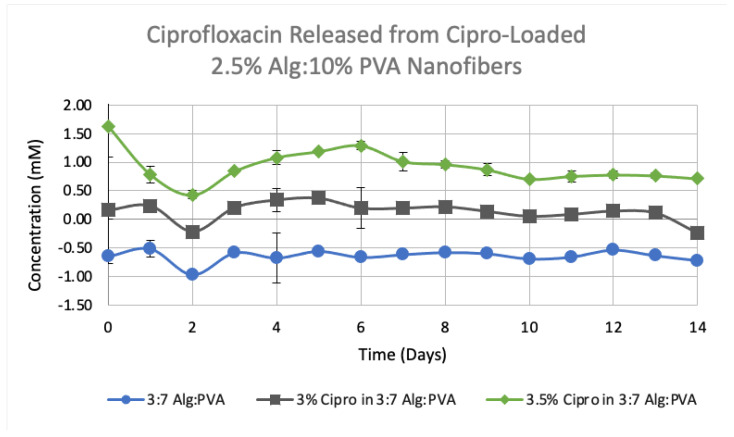
Comparison of average ciprofloxacin release over 14 days from 3–3.5% (*w/v*) Cipro-loaded 3:7 2.5% Alg:10% PVA nanofibers and control, non-loaded 3:7 2.5% Alg:10% PVA nanofibers (*n* = 3, *p* ≤ 0.001). All fiber scaffolds were 1.5 cm × 1.5 cm. A standard deviation for each time point is given.

An additional study was conducted to compare the release rate of ciprofloxacin from drug-loaded scaffolds containing different amount of the antibiotic (3.0% vs, 3.5% (*w/v*)). All scaffolds in this study measured 1.5 cm × 1.5 cm. While both drug-loaded samples released ciprofloxacin over time, it was found that the 3.5% (*w/v*) ciprofloxacin-loaded samples released more drug over the same amount of time when compared to the 3% (*w/v*) loaded sample. A *p*-test conducted at 95% confidence, indicated that the ciprofloxacin release of each drug-loaded scaffold was significantly different from the non-loaded, control fibers. Overall, it was determined that the 1.5 cm × 1.5 cm, 5% and 3.5% (*w/v*) ciprofloxacin-loaded 3:7 2.5% (*w/v*) Alg:10% (*w/v*) PVA fiber mats showed the largest amount of release (Figure 9). These results were anticipated as these samples contained the highest amounts of ciprofloxacin. We observed a sustained release of the antibiotic over the course of 14 days. This gradual release is comparable to the release profiles of ciprofloxacin from alginate/poly(ethylene oxide) nanofibers [34] and the release of metronidazole from alginate/PVA fibers [36].

The described drug release studies are compiled in Table 1 with electrospinning parameters and the average ciprofloxacin concentration released over 14 days.

## 4. Discussion

The optimal polymer solution to prepare Alg-PVA nanofibers was determined to be a 3:7 ratio of 2.5% (*w/v*) alginate and 10% (*w/v*) PVA dispensed at a rate of 7.5 µL/min, with a target distance of 100 mm, with a voltage of 30 kV. This formulation combined with these electrospinning conditions produced smooth, uniform fibers with an average diameter of 185.1 nm. Agarose’s low water solubility hindered the creation of nanofibers containing both alginate and agarose. However, thin fibers were formed using a 1:2:1 ratio of 2.5% Alg:10% PVA:0.5% Ag (*M* = 122.5 nm). These Alg-PVA-Ag fibers still showed beading and thus were not drug–loaded. Once further experimentation identifies a set of finalized parameters for the preparation of Alg-PVA-Ag fibers, these fibers will be loaded with ciprofloxacin and drug release data for these fiber formulations collected.

Ciprofloxacin was successfully introduced into 3:7 2.5% (*w/v*) Alg:10% (*w/v*) PVA nanofibers in various concentrations and multiple release studies were performed. The lower ciprofloxacin concentrations showed decreased beading and webbing on the samples, producing more uniform fibers with significantly larger diameters. Regardless of ciprofloxacin concentration, drug-loaded fibers had a higher standard deviation of the average fiber diameter as compared to the control, non-loaded fibers. Our fiber diameters and their corresponding standard deviations were consistent with those reported for other alginate-based and drug-loaded nanofibers in the literature. The increase in the thickness of our 3 and 3.5% (*w/v*) ciprofloxacin-loaded fibers diameters was similar to that of other drug-loaded fibers that have been reported. While it is possible to load more of the drug into scaffold, lower concentrations were used in these studies due to the more uniform fiber architecture of the drug-loaded mats. It was found that the 3.5% (*w/v*) ciprofloxacin-loaded fibers released the drug more effectively over time as compared to the 3% or 5% (*w/v*) ciprofloxacin-loaded fibers and the release profile was consistent with that reported in the literature.

In the future, fluorescently tagged ciprofloxacin will be electrospun into alginate and PVA fiber scaffolds to verify the location of the drug molecules in the polymer mat via confocal imaging. Towards the overarching goal of understanding the impact of co-polymers and innate polymer charges on drug release rates, ciprofloxacin will be electrospun with positively charged chitosan and PVA to determine if the inherent charges on natural polymers influences the release rate of therapeutics. We anticipate investigating the release of other drugs from natural polymer-based fibers including large therapeutics such as proteins. The release rates of each therapeutic from the corresponding fiber formulations will be compiled into and expanded release profile catalog. Furthermore, the antibacterial properties of these materials will be investigated as well as cell responses to these novel nanofibers to observe their antibacterial capabilities [37]. Long term, we anticipate that these drug-loaded fibers will have applications within the biomedical field as have other nanomaterials, including utilization as drug delivery vehicles and antibiotic-containing wound dressings [38].

## Figures and Tables

**Table 1:** Compilation of Alg-PVA Release Study Runs.

Polymer Ratio	Polymer Formulation	Cipro Percentage (*w/v*)	Rate (μL/min)	Height (mm)	Voltage (kV)	Volume Dispersed (mL)	Average Cipro Release ^1^ (mM)
3:7	2.5% Alg:10% PVA	0	9.8	100	16	1	0
3:7	2.5% Alg:10% PVA	3.0	9.8	100	16	1	0.134
3:7	2.5% Alg:10% PVA	3.5	9.8	100	16	1	0.920
3:7	2.5% Alg:10% PVA	5	9.8	100	16	1	0.924

^1^ Averaged over 14 days.

## Data Availability

Not applicable.
